# Progress of the Principle of Patients' Payments

**Published:** 1920-10-30

**Authors:** 


### The Hospital

The Workers' Newspaper of Administrative Medicine and Institutional Life, Administration, National Insurance and Health.

No. 1795, Vol. LXIX. SATURDAY, OCTOBER 30, 1920. PRICE TWOPENCE

### PROGRESS OF THE PRINCIPLE OF PATIENTS' PAYMENTS.

We are informed by Viscount Hambleden, whose letter appears on another page, that the London Committee of the British Hospitals Association have been considering the question of patients' payments, and advise that the time has arrived when the principle of requiring patients to contribute towards the cost of their maintenance should be recommended for general adoption in London. In view of the recent organisation of these payments at King's College Hospital, Lord Hambleden will have been able to guide his Committee as to the possibilities of the movement.

Of course, the London Committee speak for London only. This is not only proper, but wise, for one of the chief of the many things in which London hospitals differ from provincial hospitals is in this matter of patients' payments, as we have pointed out more than once. In an industrial district, where a hospital relies almost entirely upon its own district for support, and receives its patients from a defined area, it is plain that its position is altogether different from a London institution which has no particular district from which it exclusively draws its patients, and to which it can look for the chief means of support. In the former instance the people of the district, as regards the hospital, have responsibilities, as well as obligations, and, when they worthily carry out those responsibilities, they often have a voice in management of their local institution. Therefore it is not surprising to gather, from the information at our disposal, that the subscribers of the Metropolitan hospitals, speaking generally, are much in favour of the principle of patients' payments, while those in the provinces, more particularly in industrial districts, are not so unanimous. After all, it is largely a matter of getting money to run the hospital, and if the employers and employees of a district prefer to do this without resorting to payment by patients, well and good.

But we think it must be one thing or the other. We do not at all like the idea brought forward at the monthly committee meeting of Queen Mary's Hospital for the East End that a subscriber's letter should go towards exempting a patient from making a payment. The letter system is antiquated and thoroughly bad. It leads to petty patronage and sets up the danger that a patient may have precedence for another reason than his medical condition, which should be always the paramount factor. Anything which serves to strengthen the "letter" system will, we feel certain, secure the condemnation of all who have the best interests of the voluntary system at heart.

The British Hospitals Association find, as a result of inquiry, that practically all London hospitals are now, in one way or another, asking the patients to contribute according to their means, and as a result a considerable revenue is being derived from this source. The institution of a flat rate is not favoured as a rule, but it is left to experience to show whether the best results are obtained by adopting this method or that of assessment according to means. The expression "best results," of course, has a purely financial meaning. To delay admission of a patient who needs early treatment while the relatives are canvassing friends and relief agencies to make up what they can afford to what is demanded would be one of the worst results of a flat rate.

We are greatly in favour of conducting hospital finance on a budget basis. We must discover what revenue we are likely to require, then compute what support may be expected from the central funds, from investments, from subscriptions and donations, from other regular sources, and what remains must come from the patients, from the relatives, from the insurance companies interested in the patients, from Poor-law Guardians, or from whoever is responsible. We suggest that when the estimated amount is known it shall be spread over the available beds. If it is found that, say, two guineas per bed must come from patients or their friends, then an average of two guineas must be aimed at. Those better off must pay a share of those not so well provided with money or friends. A frequent review must be made to see that the average is being kept up, otherwise the aggregate sum aimed at will sag away and the hospital will once more be in difficulties.

In this work the almoners will be found particularly useful and they will secure a just assessment and arrange for collection of fees. We have reason to believe that almoners are not in love with the system, and, possibly in accordance with the maxim that it is better to give than to receive, are inclined to think that their main work concerns spending rather than getting. Nobody would wish to interfere with their mission of putting the coping-stone on the work of the doctors and nurses by means of efficient after-care help and of other methods of ensuring that what is given by the hospital is neither wasted nor marred for need of their auxiliary labours. All will agree that such functions must proceed unimpeded, but just now we have need of the trained judgment for these officers in matters of ascertaining the patient's resources of self-help and of their knowledge of agencies which will, if necessary, help the patient to meet his share of the expense of maintenance and treatment. Almoners must move with the times in this respect, because we cannot do without their help, trained under the old conditions, unless we seek a new kind of almoner trained in the light of newer methods.

Unfortunately, it looks as if a period of considerable unemployment is close upon us. This will affect the general prosperity of the patient, and the agencies which would help him will possibly have urgent calls in other directions. The coming months will, therefore, not be a fair test of the financial success of the system, but of the soundness of the principle of patients' payments, at least as regards the hospitals of great cities, few can have a doubt.

